# Analysis of *FCGR3A*-p.176Val variants in women with recurrent pregnancy loss and the association with CD16a expression and anti-HLA antibody status

**DOI:** 10.1038/s41598-023-32156-9

**Published:** 2023-03-30

**Authors:** Denise H. J. Habets, Salwan Al-Nasiry, Sietse Q. Nagelkerke, Christina E. M. Voorter, Marc E. A. Spaanderman, Taco W. Kuijpers, Lotte Wieten

**Affiliations:** 1 Department of Obstetrics and Gynecology, Maastricht University Medical Centre, Maastricht, The Netherlands; 2 Department of Transplantation Immunology, Maastricht University Medical Centre, Maastricht, The Netherlands; 3 GROW School for Oncology and Reproduction, Maastricht University, Maastricht, The Netherlands; 4 Department of Blood Cell Research, Sanquin Research and Landsteiner Laboratory, Amsterdam University Medical Centre, University of Amsterdam, Amsterdam, The Netherlands; 5 Department of Pediatric Immunology, Rheumatology and Infectious Diseases, Emma Children’s Hospital, Amsterdam University Medical Centre, University of Amsterdam, Amsterdam, The Netherlands

**Keywords:** Developmental biology, Immunology

## Abstract

Natural Killer (NK) cells have been implicated in recurrent pregnancy loss (RPL). The p.Val176Phe (or Val158Phe) Single Nucleotide Polymorphism (SNP) in the *FCGR3A* gene encoding the FcγRIIIA or CD16a receptor has been associated with an enhanced affinity for IgG and stronger NK-mediated antibody-dependent cellular cytotoxicity. We hypothesized that the presence of at least one p.176Val variant associates with RPL and increased CD16a expression and alloantibodies e.g., against paternal human leukocyte antigen (HLA). In 50 women with RPL, we studied frequencies of the p.Val176Phe *FCGR3A* polymorphisms. Additionally, CD16a expression and anti-HLA antibody status were analyzed by flowcytometry and Luminex Single Antigens. In woman with RPL, frequencies were: 20% (VV), 42% (VF) and 38% (FF). This was comparable to frequencies from the European population in the NCBI SNP database and in an independent Dutch cohort of healthy women. NK cells from RPL women with a VV (22,575 [18731-24607]) and VF (24,294 [20157-26637]) polymorphism showed a higher expression of the CD16a receptor than NK cells from RPL women with FF (17,367 [13257-19730]). No difference in frequencies of the *FCGR3A*-p.176 SNP were detected when comparing women with or without class I and class II anti-HLA antibodies. Our study does not provide strong evidence for an association between the p.Val176Phe *FCGR3A* SNP and RPL.

## Introduction

Recurrent Pregnancy Loss (RPL) is a devastating problem for couples trying to conceive. Despite observed clinical associations, the current knowledge on etiological factors in RPL is still incomplete and more than 50% of cases remains unexplained^1^. As the fetus is genetically semi-allogenic to the mother, tight regulation of immune reactivity is essential for the proper establishment and continuation of successful pregnancy^2^. Emerging evidence now suggests that dysregulation of Natural Killer (NK) cell function could be responsible for several cases of unexplained RPL^3^.

NK cells are innate lymphocytes and peripheral blood NK cells (pNK) are primarily known as potent killers of virally-infected or tumor cells. NK cells kill through the release of granzyme and perforin containing granules or via death receptors^4^. Moreover, they can produce cytokines contributing to Th1 polarization and CD8 T cell function^5^. Two important lineages of NK cells have been described that can be classified according to the expression level of CD56: CD56^dim^ NK cells predominantly express the FcγRIIIA receptor also referred to as CD16a and constitute around 90% of NK cells in peripheral blood and mediate cytotoxicity. Conversely, CD56^bright^ NK cells lack expression of CD16a and while they are mostly known for their capacity to produce cytokines, they are poorly cytotoxic and form merely 5–10% of NK cells in peripheral blood^6^. In the secretory phase of the menstrual cycle and during early pregnancy, CD56^bright^CD16a^negative^ NK cells constitute the predominant lymphocyte subset present in the non-pregnant endometrial or pregnant decidual layer of the uterus^7^. In contrast to their killing function in peripheral blood, uterine NK (uNK) cells regulate trophoblast invasion and promote placental vasculature hence contributing to adequate placentation^8,9^. The considerable interest in the role of uNK cells in pregnancy complications, particularly in RPL, indicates the perceived importance of these cells in early pregnancy. Although some controversy still exists, overall results indicate that RPL is associated with several abnormalities in NK cell number and activation^10^.

NK cell activation is controlled by a broad panel of inhibitory- and activating receptors, and the balance between signaling via these receptors determines the level of NK cell activation^11^. CD16a is one of the most powerful activating receptors on NK cells and it enables NK cells to respond to antibody-coated target cells and to exert antibody-dependent cellular cytotoxicity (ADCC)^12^. The process of ADCC starts with the binding of an antibody to its cognate antigen expressed on the target cell surface. The Fc domain of these antibodies is then bound by CD16a expressed on NK cells, triggering the release of cytotoxic granules and subsequent lysis and death of the target cell^13^. The strength of NK cell mediated ADCC is determined by factors like the isotype- or fucosylation status of the antibody^14,15^. Moreover, the rs396991 c.526G > T single nucleotide polymorphism (SNP) in the *FCGR3A* gene, results in expression of either valine or phenylalanine at amino acid position 176 (also known as position 158 in the mature protein excluding signal peptides) and has been reported to influence human IgG_1_ binding and ADCC activity^16^. Functional studies have shown that the presence of a homozygous p.176-valine (VV) results in a CD16a receptor with a higher affinity for IgG1 and IgG3 antibodies compared to the homozygous p.176-phenylalanine genotype (FF) and the triggering of NK cell activation or ADCC at a lower antibody concentration^17–20^. Moreover, two of these studies also suggested a gene dose effect since NK cells from heterozygous individuals showed an intermediate response^17,18^. ADCC is a major mechanism of action of therapeutic monoclonal antibodies (mAbs) such as rituximab and a higher ADCC response to rituximab as well as increased CD16a expression have been reported to correlate with the genotypes encoding a p.176-valine in the *FCGR3A* gene^18^. Nonetheless, another study did not find a relation between the p.176 polymorphism and CD16a expression on NK cells^21^. Multiple clinical studies in patients with various types of cancers, showed that homozygosity (VV), or in some studies the presence of at least one high affinity allele (Vx), was associated with an improved response to treatment with therapeutic antibodies like rituximab, trastuzumab or cetuximab^16^.

The percentage of uNK cells that express CD16a under physiological conditions is low, however, an elevated percentage of CD16a positive cells has been observed in woman with pregnancy complications^19,20^. In a mouse study, uNK cells have been shown to mediate ADCC against invading trophoblast cells of the fetus in an CD16a dependent manner^23^. Furthermore, 30% of women develop antibodies against paternal human leukocyte antigen (HLA) alloantigens expressed by the fetus^24^. These alloantibodies are considered a harmless phenomenon during most pregnancies primarily because the fetal cells most closely in contact with the immune system almost completely lack expression of polymorphic HLA class I and class II molecules^25^. Nevertheless, several studies suggested that these anti-HLA antibodies were associated with RPL as a higher incidence of anti-HLA antibodies has been observed in women with RPL compared to women without^26^. Although the potential underlying mechanism is not very well explored, these alloantibodies may contribute to RPL via complement fixation and/or ADCC resulting in damage of the invading trophoblast cells showing aberrant expression of polymorphic HLA molecules.

**Figure 1:**
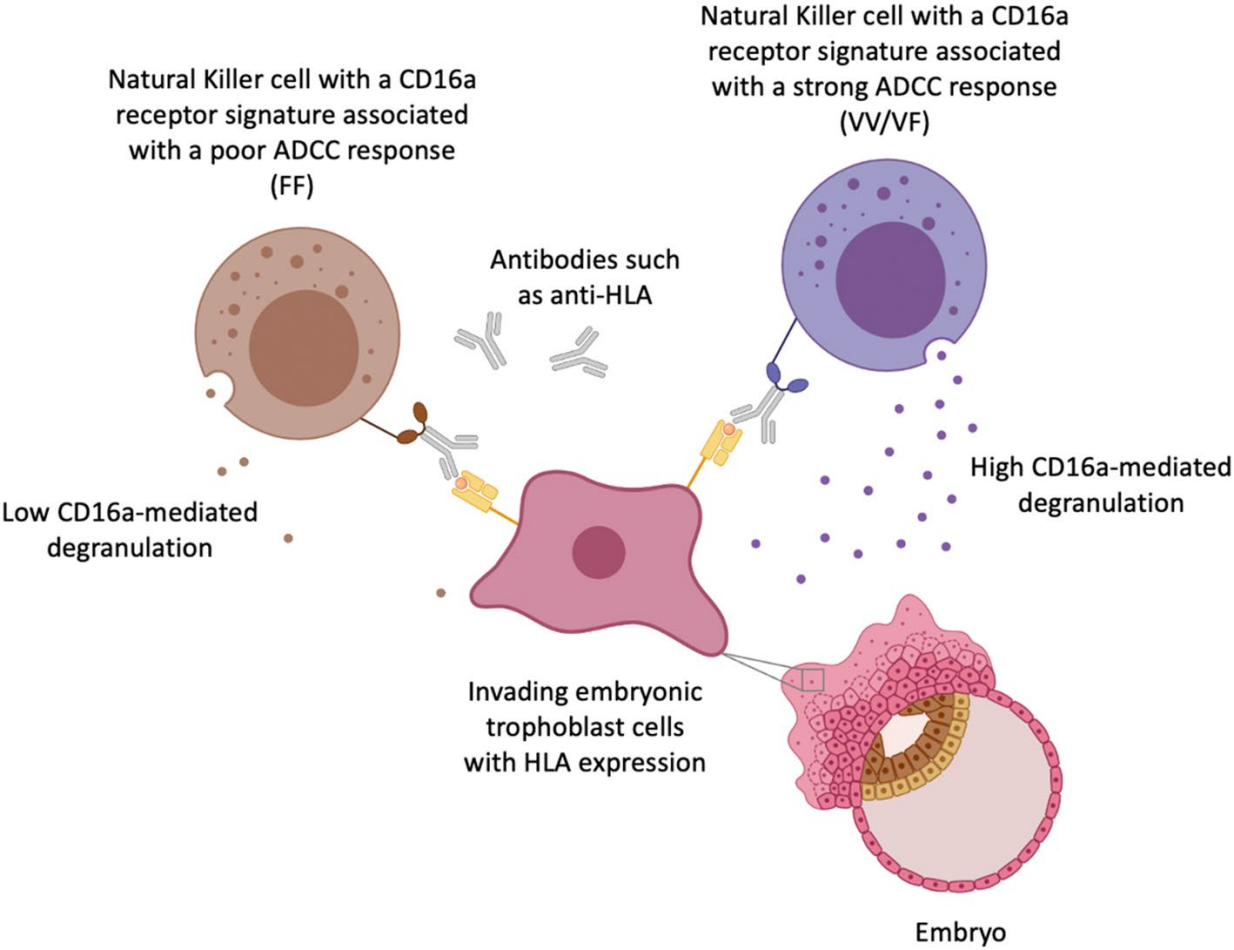
Illustrative representation of antibody dependent cellular cytotoxicity (ADCC) of Natural Killer (NK) cells in the uterus with a CD16a receptor having two phenylalanine alleles (FF) depicted in brown and a CD16a receptor having at least one valine allele (VV/VF) depicted in purple. We hypothesize that a receptor having at least one valine allele induces a stronger ADCC response and an increased CD16a-mediated degranulation capacity of NK cells against the trophoblast in women with recurrent pregnancy loss (RPL).

Given the possible impact of *FCGR3A* polymorphism on the strength of the ADCC response, we hypothesized that in women with RPL having at least one *FCGR3A*-p.176Val variant enhances the ADCC response, see Fig. 1. As such, we identified *FCGR3A*-p.176 polymorphisms in women with RPL. We compared these frequencies to the frequencies in the NCBI single nucleotide polymorphisms database and an independent cohort of healthy women. In addition, we sought to delineate differences in NK cell surface CD16a expression in women with RPL with a VV, VF and FF SNP. Since we expect the effect of *FCGR3A* polymorphism to be most relevant in women with antibodies against fetal alloantigens, we also determined HLA antibody status in women with RPL and studied distribution of the polymorphic subgroups.

## Results

### Baseline characteristics of women with RPL

Women with RPL were on average 31 years old, had on average 5 pregnancies, 4 pregnancy losses and no live birth (Table 1).

**Table 1:** Baseline characteristics of study population.

	RPL (n = 50)
Age	31.2 ± 3.6
Gravida	5 ± 2
Para	0 ± 1
Pregnancy losses	4 ± 2

Baseline characteristics of study population, data are presented as average ± standard deviation.

### Frequencies of *FCGR3A*-p.176 genotypes in women with RPL are similar to frequencies in a European cohort and an independent cohort of healthy women

To study frequencies of the p.Val176Phe *FCGR3A* genotype in women with RPL, single-nucleotide changes in position 526 (G → T) were detected by sanger sequencing. Based on position 176, the following subgroups of *FCGR3A* genotypes were identified: valine/valine (VV), valine/phenylalanine (VF) and phenylalanine/phenylalanine (FF). Genotypes were subsequently identified as (VV/VF and FF) subgroups. The frequencies were as follows: 31 out of 50; 62% (VV/VF) and 19 out of 50; 38% (FF) for women with RPL (Table 2). When genotypes were identified as (VF/FF) and (VV) subgroups the frequency was 40 out of 50; 80% for (VF/FF) and 10 out of 50; 20% for (VV). No significant deviation from the Hardy–Weinberg expectation was observed.

**Table 2:** Frequencies of *FCGR3A*-p.176 genotypes in women with RPL, a cohort of healthy women and a European cohort.

	RPL (n = 50)	Healthy female cohort (n = 164)	*P*	European population (n = 32,758) (%)
VV/VF	62.0% (31/50)	53.0% (87/164)	0.265	52.4
FF	38.0% (19/50)	47.0% (77/164)		47.6
VF/FF	80.0% (40/50)	86.6% (142/164)	0.253	90.4
VV	20.0% (10/50)	13.4% (22/164)		9.6
VV	20.0% (10/50)	13.4% (22/164)	0.374	9.6
VF	42.0% (21/50)	39.6% (65/164)		42.8
FF	38.0% (19/50)	47.0% (77/164)		47.6

Frequencies of *FCGR3A*-p.176 genotypes were based on the valine and phenylalanine SNP on amino acid position 176 for RPL women, healthy women and calculated from ALFA Allele Frequency (dbSNP) for a European population.

Since no matched control group was available, the frequencies of a general European cohort were obtained from the NCBI single nucleotide polymorphisms database (dbSNP) and they were as follows: 52.4% (VV/VF) and 47.6% (FF) and additionally 90.4% (VF/FF) and 9.6% (VV) (Table 2). In addition, frequencies were used from a Dutch cohort of healthy females that was genotyped by MLPA for a previously published study. The frequencies for this cohort were comparable to the European cohort: 87 out of 164; 53% (VV/VF), 77 out of 164; 47% (FF) and 142 out of 164; 86.6% (VF/FF), 22 out of 164; 13.4% (VV) (Table 2). No significant deviation from the Hardy–Weinberg expectation was observed.

To enable statistical comparison of both cohorts, we first validated that the MLPA method used for typing of the healthy female cohort and the SBT method used for typing of the RPL cohort gave similar results. To do this, 5 women with RPL were genotyped for *FCGR3A* p.V176F by MLPA which gave identical genotypes as was obtained when the samples were analyzed by SBT. A statistical comparison of the RPL and the healthy control cohort subsequently showed that there was no significant difference between women with RPL and healthy women (*P* = 0.265) when comparing (VV/VF) and (FF) subgroups. Furthermore, there was no significant difference between women with RPL and the healthy control cohort when comparing (VF/FF) versus (VV) (*P* = 0.253). In addition, there was no significant difference (*P* = 0.374) when frequencies were compared for VV (20%; 10 out of 50), VF (42%; 21 out of 50) and FF (38%; 19 out of 50) in women with RPL, versus respectively 13.4%; 22 out of 164 (VV), 39.6%; 65 out of 164 (VF) and 47%; 77 out of 164 (FF) in healthy women (Table 2).

### Analysis of the influence of the p.V176F polymorphism on CD16a positive NK cells

**Figure 2:**
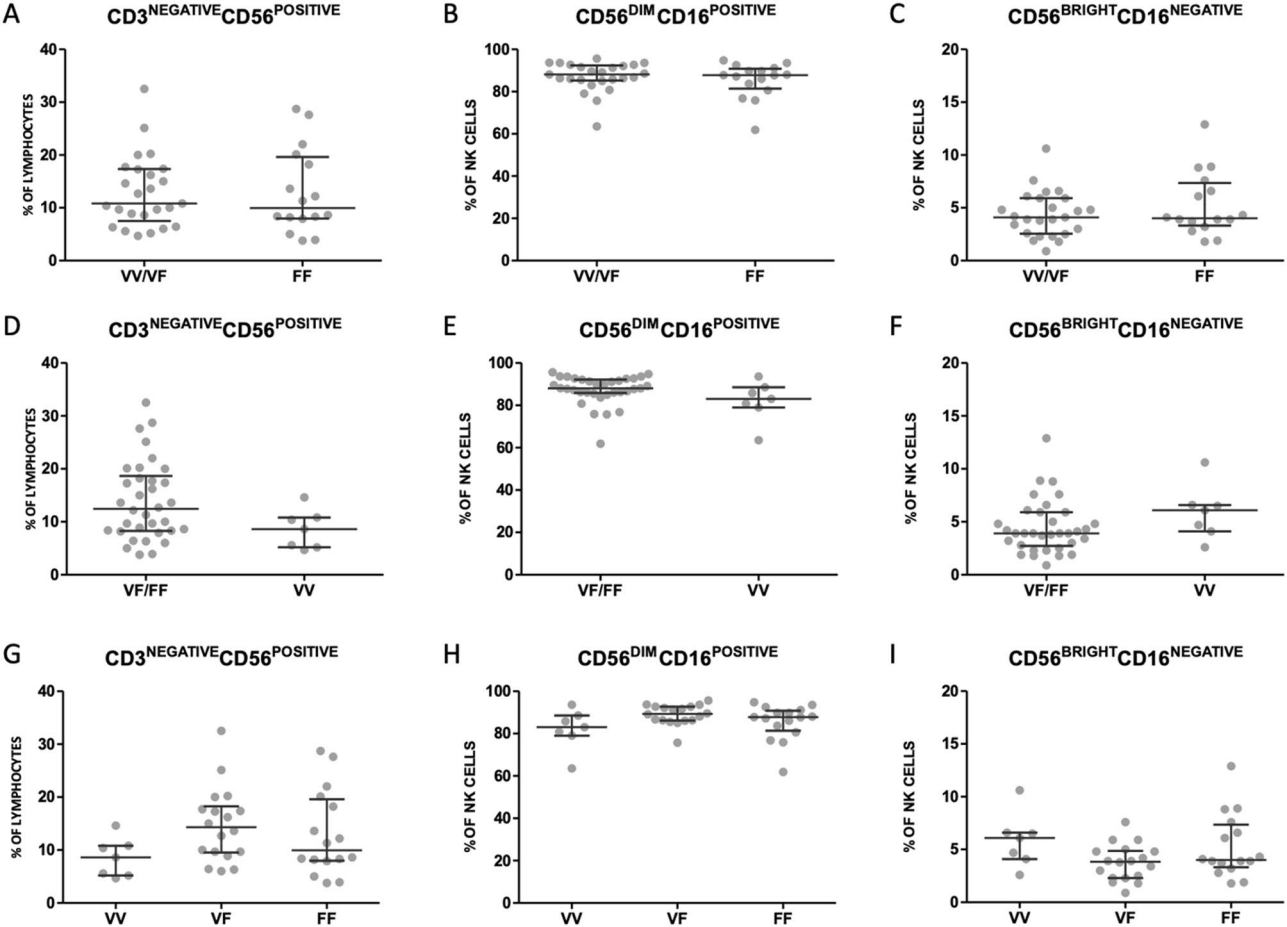
Frequencies of NK cell subsets in women with RPL genotyped for CD16a. Percentage of total CD3^negative^CD56^positive^ NK cells from lymphocytes (**A**), CD56^dim^CD16a^positive^ NK cells (**B**) and CD56^bright^CD16a^negative^ NK cells (**C**) as measured by flow cytometry in women whose genotyping demonstrated *FCGR3A*-p.176 at least one valine allele (VV/VF) versus (FF) (**A**/**B**/**C**) or at least one phenylalanine allele (VF/FF) versus (VV) (**D**/**E**/**F**) or VV, VF and FF (**G**/**H**/**I**) respectively. Dots depict individuals, lines depict median and inter quartile range per group.

In addition to qualitative differences, the p.V176F SNP has been shown to result in quantitative differences in the level of receptor expression on NK cells. To study if these quantitative differences also occurred in our cohort of RPL women, the percentage of CD16a positive NK cells and the level of expression were determined by flowcytometry (gating strategy supplementary file) and compared between VV/VF and FF subgroups and additionally between VF/FF and VV subgroups of women with RPL. First, we compared distribution of total NK cells, defined as CD3^negative^CD56^positive^ and of the two main NK cell subsets; the CD56^bright^CD16a^negative^ mostly known as cytokine producers and the cytotoxic CD56^dim^CD16a^positive^. No significant differences were found between the VV/VF and the FF subgroup when comparing percentage of total CD3^negative^CD56^positive^ NK cells (VV/VF 10.8 [7.5-17.4] versus FF 10.0 [8.0-19.6] *P* = 0.708), the percentage of CD56^dim^CD16a^positive^ NK cells (VV/VF 88.1 [85.3-92.4] versus FF 87.8 [81.5-90.8] *P* = 0.612), or the percentage of CD56^bright^CD16a^negative^ NK cells (VV/VF 4.1 [2.6-5.9] versus FF 4.0 [3.3-7.4] *P* = 0.470) (Fig. 2A–C).

Second, no significant differences were found between VF/FF and VV subgroups when comparing percentage of total CD3^negative^CD56^positive^ NK cells (VF/FF 12,5 [8.3-18.6] versus VV 8,6 [5.2-10.8] *P* = 0.080), the percentage of CD56^dim^CD16a^positive^ NK cells (VF/FF 88.1 [85.9-92.2] versus VV 83.0 [79.0-88.5] *P* = 0.096), or the percentage of CD56^bright^CD16a^negative^ NK cells (VF/FF 3.9 [2.7-5.9] versus VV 6.1 [4.1-6.6] *P* = 0.099) (Fig. 2D–F).

Additionally, there was no significant difference in percentage of total NK cells (*P* = 0.120) when percentages were compared between VV (8.6 [5.2-10.8]), VF (14.3 [9.5-18.3]) and FF (10.0 [8.0-19.6]). Neither when percentage of CD56^dim^CD16a^positive^ NK cells (*P* = 0.123); VV (83.0 [79.0-88.5]), VF (89.3 [86.2-92.7]), FF (87.8 [81.5-90.8), nor when percentage of CD56^bright^CD16a^negative^ NK cells (*P* = 0.094); VV (6.1 [4.1-6.6]), VF (3.9 [2.3-4.9), FF (4.0 [3.3-7.4]) was compared between VV, VF and FF (Fig. 2G–I).

**Figure 3:**
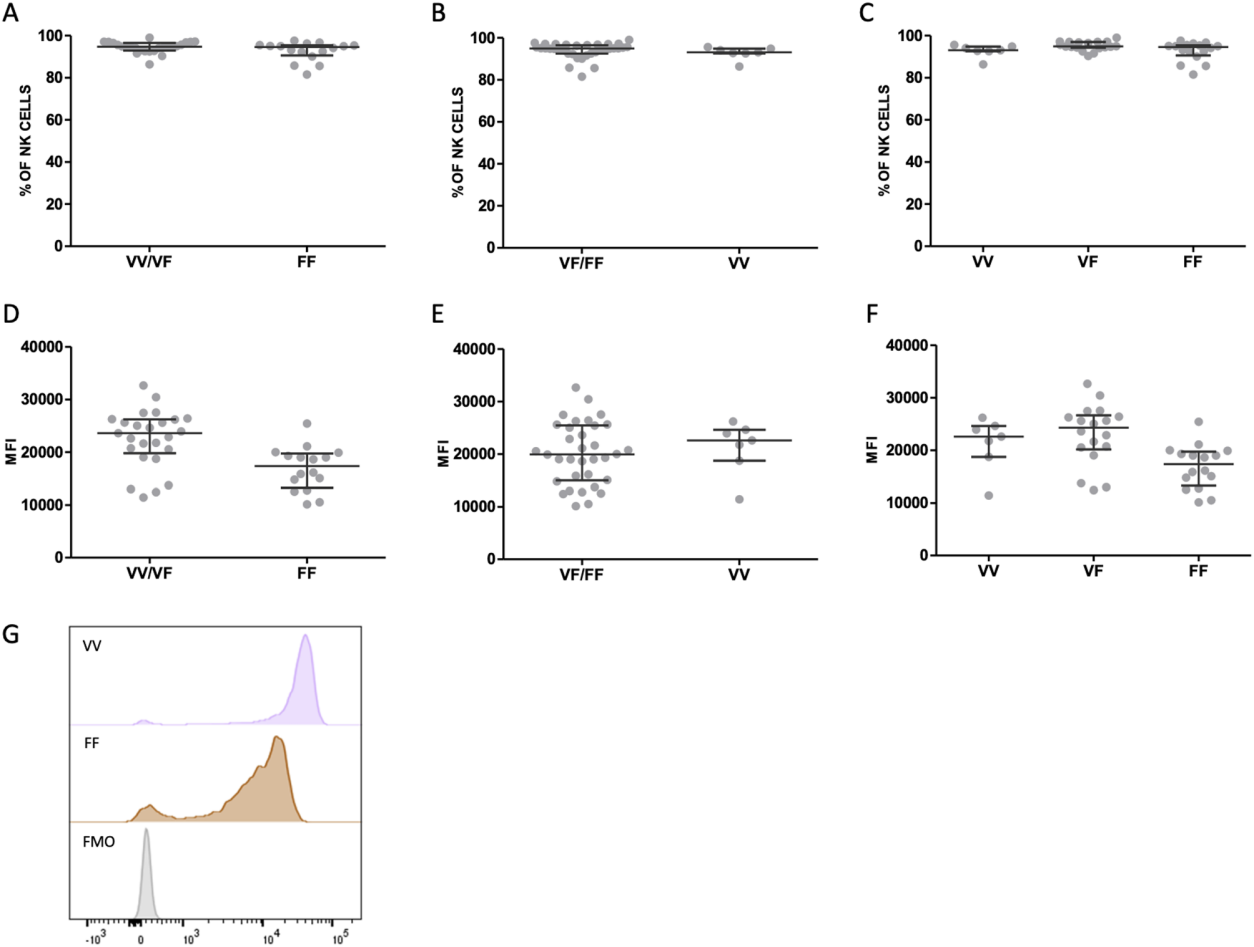
CD16a expression on NK cells in women with RPL genotyped for CD16a. Percentage of CD16a positive cells of total CD3^negative^CD56^positive^ NK cells (**A**/**B**/**C**) and MFI (**D**/**E**/**F**) normalized by FMO of CD16a expression measured by flow cytometry in women with at least one valine (VV/VF) versus (FF) *FCGR3A*-p.176 genotypes (**A** and **D**) or at least one phenylalanine allele (VF/FF) and (VV) genotypes (**B** and **E**) or VV, VF and FF genotypes (**C** and **F**). Dots depict individuals, lines depict median and interquartile range per group, *P < 0.05. Representative histogram of CD16a expression on NK cells (G) depicting FMO (no anti-CD16a) in grey and CD16a expression for the (VV) genotype in purple and the (FF) genotype in brown.

Next, the percentage of CD16a on all NK cells, irrespective of the expression level of CD56, was compared between the VV/VF and FF groups among women with RPL. No significant difference (*P* = 0.329) was observed between both groups (VV/VF 94.8 [93.0-96.7]) versus FF: 94.6 [90.7-95.5]) (Fig. 3A). Similarly, there was no significant difference between polymorphic subgroups when percentages of CD16a on total CD3^negative^CD56^positive^ NK cells (*P* = 0.176) were compared between (VF/FF): 95.0 [92.5-96.6] and (VV): 93.1 [92.6-94.9] (Fig. 3B). Moreover, there was no significant difference between subgroups when percentages of CD16a on total CD3^negative^CD56^positive^ NK cells (*P* = 0.116) were compared for VV (93.1% ([92.6-94.9]), VF (95.0% [94.3-97.0]) and FF (94.6% [90.7-95.5]) (Fig. 3C).

However, we observed a higher MFI for CD16a in RPL women with the VV/VF genotype (23575 [19782-26220]) versus the FF genotype (17367 [13257-19730]) (*P* = 0.001, Fig. 3D) but not in women in the (VF/FF) group (19924 [14971-25454]) versus the (VV) group (22575 [18731-24607]) (*P* = 0.603), Fig. 3E). In line with those data, the CD16a MFI was higher for RPL women with the VV or VF genotype than for RPL women with the FF genotype: MFI for VV was 22575 [18731-24607], for VF was 24294 [20157-26637] and for FF was 17367 [13257-19730] (*P* = 0.003) (Fig. 3F). All MFI values were normalized with a fluorescent minus one (FMO) (Fig. 3G).

### No obvious association between HLA antibody status and frequencies of *FCGR3A*-p.176 genotypes

**Figure 4:**
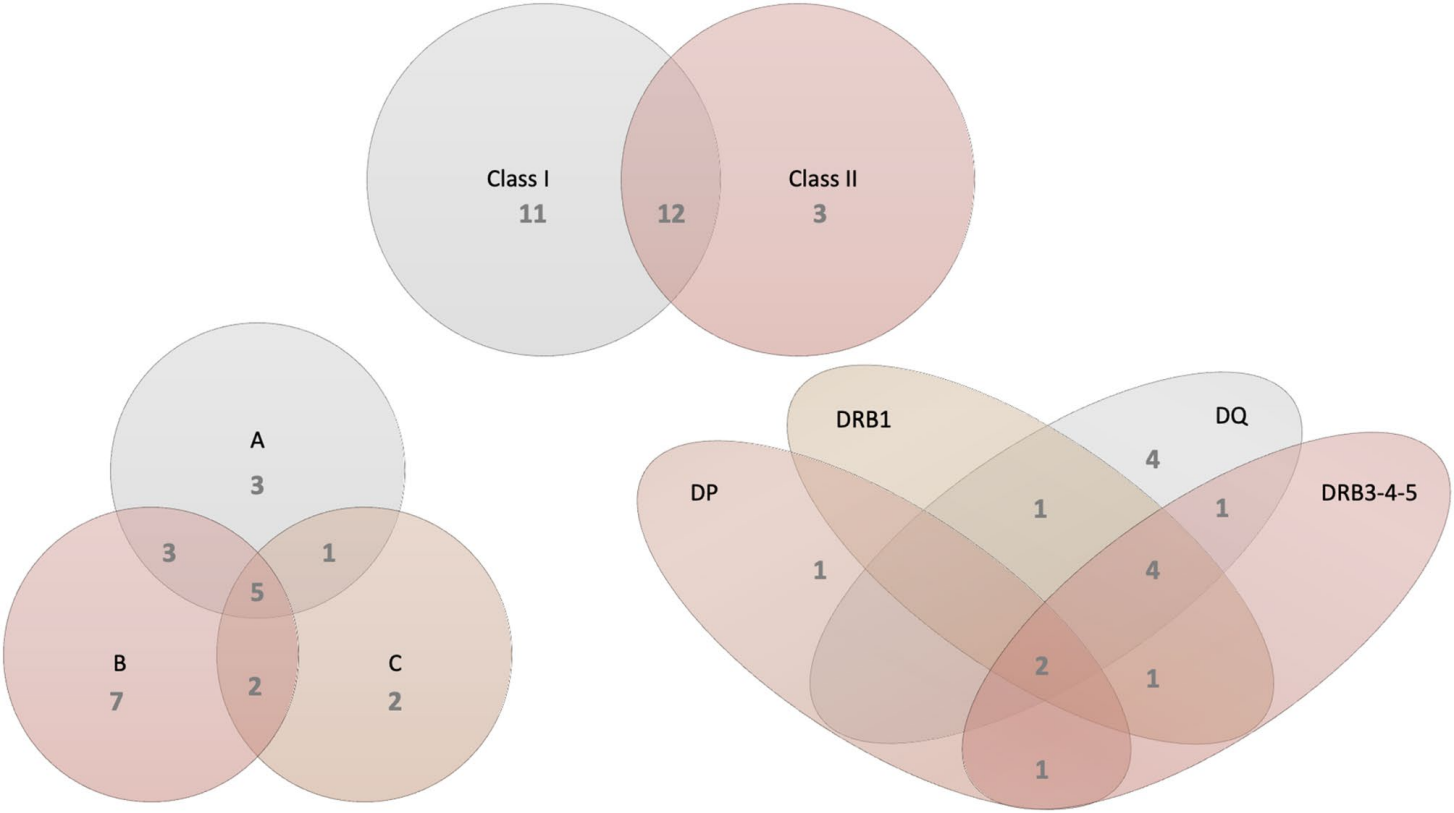
Specificity of anti-HLA antibodies. Anti-HLA antibodies, including HLA-A, B, C, DP, DQ, and DR, were detected in sera of 32 women with RPL. Specificity and number of individuals that were positive for anti-HLA antibodies are indicated in a Venn diagram representation.

The impact of the *FCGR3A*-p.176 polymorphism will primarily be relevant in combination with maternal IgG alloantibodies with the potential to trigger NK cell mediated ADCC against fetal cells expressing paternal alloantigens. In the transplantation setting, the highly polymorphic family of HLA molecules (HLA-A/-B/C and HLA-DR/-DQ/-DP) represents the most important alloantigens and anti-HLA antibodies can provoke complement- and/or cell-mediated cytotoxicity against cells of the allograft^27^. Even in normal pregnancies, approximately 30% of women develop anti-HLA antibodies^24^. To obtain initial evidence for a potentially synergistic role of anti-HLA antibodies and *FCGR3A*-p.176 polymorphism, we determined, as a small pilot, the presence and specificity of anti-HLA antibodies by Luminex Single Antigens in 32 women from our RPL cohort. Anti-HLA antibodies were present in 26 out of 32 women (Fig. 4). Antibodies were directed against HLA class I in 11/32 women, against HLA class II in 3/32 women or against both, class I and class II in 12/32 women and we further defined specificity of the HLA class I and -class II antibodies found in the 32 tested women with RPL (Fig. 4). Out of 23 women who had antibodies against class I, 7 had antibodies against HLA-B, 3 against HLA-A, 2 against HLA-C, 1 against HLA-A + C, 2 against HLA-B + C, 3 against HLA-A + B and 5 against HLA-A + B + C. Out of 15 women who had antibodies against class II, 1 had antibodies against HLA-DP, 4 against HLA-DQ, 1 against HLA-DQ + DRB1, 1 against HLA-DQ + DRB3/4/5, 4 against HLA-DQ + DRB1 + DRB3/4/5, 1 against HLA-DRB1 + DRB3/4/5, 1 against HLA-DP + DRB3/4/5 and 2 against HLA-DP + DQ + DRB1 + DRB3/4/5 (Fig. 4).

Next, we determined the frequencies of (VV/VF) and (FF) polymorphic subgroups of RPL women and compared them between women with and without antibodies. Frequencies of VV/VF in women with antibodies were 57,7% (class I and/or class II Ab positive), 60,9% (class I Ab positive), 46,7% (class II Ab positive) compared to 66,7%% in women who completely lacked anti-HLA antibodies, 55,5% in women without class I antibodies and 70,6% in women without HLA class II antibodies (Table 3). In this small group of patients, we did not observe a significant difference in frequencies of the p.Val176Phe genotypes (VV/VF versus FF) based on antibody status (class I and/or class II (*P* = 0.687), class I (*P* = 0.783) and class II (*P* = 0.169)). Additionally, we determined the frequencies of (VF/FF) and (VV) polymorphic subgroups of RPL women and compared them between women with and without antibodies in Table 3, showing that there was no significant difference in frequencies of the VF/FF versus VV genotypes based on antibody status (class I and/or class II (*P* = 0.063), class I (*P* = 0.186) and class II (*P* = 0.100)). Likewise, we did not observe a difference between women with- or without anti-HLA antibodies in frequencies of the VV, VF and FF genotypes (class I and/or class II (*P* = 0.083), class I (*P* = 0.285) and class II (*P* = 0.190)) (Table 3). Due to the low power, we did not evaluate CD16a in groups defined based on specificity of the anti-HLA antibodies.

**Table 3:** Frequencies of *FCGR3A*-p.176 genotypes in RPL women with or without class I and class II antibodies.

	Class I			Class II			Class I and/or class II		
	+ (n = 23)	− (n = 9)	*P*	+ (n = 15)	− (n = 17)	*P*	+ (n = 26)	− (n = 6)	*P*
VV/VF	60.9% (14/23)	55.5% (5/9)	0.783	46.7% (7/15)	70.6% (12/17)	0.169	57.7% (15/26)	66.7% (4/6)	0.687
FF	39.1% (9/23)	44.5% (4/9)		53.3% (8/15)	29.4% (5/17)		42.3% (11/26)	33.3% (2/6)	
VF/FF	86.9% (20/23)	66.7% (6/9)	0.186	93.3% (14/15)	70.6% (12/17)	0.100	88.5% (23/26)	50% (3/6)	0.063
VV	13.1% (3/23)	33.3% (3/9)		6.7% (1/15)	29.4% (5/17)		11.5% (3/26)	50% (3/6)	
VV	13.1% (3/23)	33.3% (3/9)	0.285	6.7% (1/15)	29.4% (5/17)	0.190	11.5% (3/26)	50% (3/6)	0.083
VF	47.8% (11/23)	22.2% (2/9)		40.0% (6/15)	41.2% (7/17)		46.2% (12/26)	16.7% (1/6)	
FF	39.1% (9/23)	44.5% (4/9)		53.3% (8/15)	29.4% (5/17)		42.3% (11/26)	33.3% (2/6)	

Class I and class II anti-HLA antibodies were tested in sera from 32 RPL women by Luminex Single Antigens and represented as positive (+) when antibodies were detected or negative (−) when antibodies were not detected. Women were subsequently grouped based on *FCGR3A*-p.176 frequencies of (VV + VF) and (FF) or (VF + FF) and (VV) or *FCGR3A*-p.176 VV, VF and FF.

The detrimental effect of the presence of donor specific antibodies (DSA) against HLA has clearly been shown in the kidney transplantation setting, where in the case of a living donor especially DSA against both HLA class I and class II, and in the case of a deceased donor DSA against HLA class I or against both HLA class I and II has been associated with reduced graft survival^28^. Hence, we compared the frequencies of VV/VF versus FF polymorphic subgroups, of VF/FF versus VV subgroups and of VV, VF and FF genotypes between women without antibodies, women with only class I antibodies, women with only class II antibodies and women with both class I and class II antibodies. No significant differences were observed between the different groups (*P* = 0.527 for VV/VF versus FF, *P* = 0.145 for VF/FF versus VV and *P* = 0.310 for VV versus VF versus FF) and having no, only class I, only class II and both class I plus II antibodies (Table 4).

**Table 4:** Comparing frequencies of *FCGR3A*-p.176 genotypes in RPL women without antibodies versus women with class I antibodies versus women with class II antibodies versus women with class I and II antibodies.

	No antibodies present	Only class I antibodies	Only class II antibodies	Only class I + II antibodies	*P*
	(n = 6)	(n = 11)	(n = 3)	(n = 12)	
VV/VF	66.7% (4/6)	72.7% (8/11)	33.3% (1/3)	50% (6/12)	0.527
FF	33.3% (2/6)	27.3% (3/11)	66.7% (2/3)	50% (6/12)	
VF/FF	50% (3/6)	81.8% (9/11)	100% (3/3)	91.7% (11/12)	0.145
VV	50% (3/6)	18.2% (2/11)	0% (0/3)	8.3% (1/12)	
VV	50% (3/6)	18.2% (2/11)	0% (0/3)	8.3% (1/12)	0.310
VF	16.7% (1/6)	54.5% (6/11)	33.3% (1/3)	41.7% (5/12)	
FF	33.3% (2/6)	27.3% (3/11)	66.7% (2/3)	50% (6/12)	

Class I and class II anti-HLA antibodies were tested in sera from 32 RPL women by Luminex Single Antigens and compared between groups of women where no antibodies were present, only class I antibody were present, only class II antibody were present and only class I and class II antibodies were present. Women were subsequently grouped based on *FCGR3A*-p.176 frequencies of (VV/VF) and (FF) or (VF/FF) and (VV) or *FCGR3A*-p.176 VV, VF and FF.

## Discussion

The present study was conducted to investigate occurrence of the p.V176F polymorphism in the *FCGR3A* gene in women with RPL. Moreover, we studied the association with NK cell surface CD16a expression and performed a small pilot to explore a potential association of the p.V176F SNP and anti-HLA antibodies.

In our women with RPL the VF genotype is the most common (42%) followed by FF (38%) and VV (20%). These frequencies are in line with results from previous studies that investigated the presence of the *FCGR3A*-p.176 polymorphism and reported VF or FF as most frequent genotype in different populations including ethnic groups from the Netherlands, Great Britain, Austria, Australia, China, Africa^29^ Norway^30^, Singapore^31^ and Japan^32^ and with the calculated frequencies from the dbSNP for a European cohort. Moreover, there was no significant difference when frequencies of women with RPL were compared to a Dutch cohort of healthy females.

Though moderate in size (n = 50), our study included a well characterized and relatively homogenous cohort of women with RPL, as the risk for clinical confounders was kept to a minimum by pre-screening the women by means of the PCVS evaluation program. By doing so cases of pregnancy loss caused by abnormal parental karyotype, thrombophilia, aberrant endocrine factors and abnormalities in ultrasound examination were excluded. Unfortunately, obstetric history was not recorded in the healthy women and no additional blood samples were available from these women for analysis of CD16a expression and anti-HLA antibody status, making it impossible to attribute differences to previous successful or unsuccessful pregnancies.

We observed a higher MFI for CD16a on NK cells from RPL women with at least one valine allele (VV/VF) (median 23,575) than on NK cells from RPL women with the homozygous phenylalanine (FF) genotype (median 17,367). However, there was no significant difference in the percentage of NK cells expressing CD16a. In our study, we used the REA423 Miltenyi antibody that recognizes the same epitope as the frequently used anti-CD16a clone 3G8. By using multiple anti-CD16a clones binding to different epitopes, three studies concluded that differences in CD16a expression levels between the p.Val176Phe variants were the result of enhanced affinity of the 3G8 clone for *FCGR3A*-p.176V rather than a true difference in the number of molecules^20,21,33^. However, two other studies that used a 3G8 clone reported similar MFI’s for CD16a for both homozygous F and V genotypes^17,34^. Although we cannot completely rule out that the difference in CD16a MFI that we observed was caused by a difference in affinity of the antibody, our data are in line with a study that showed that the absolute number of CD16a receptors per NK cell was significantly higher, in addition to higher expression of CD16a both at mRNA expression and at cell surface expression level, in individuals who expressed VV at *FCGR3A*-p.176 versus FF^18^. Additionally, it would be relevant to take copy number variation (CNV) in the *FCGR3A* gene into account in future analysis as it has been described to correlate with CD16a expression levels^35^.

In our study and previous studies assessing the impact of the p.176 SNP, CD16a expression levels were measured on pNK cells. To study the impact on RPL, it would be relevant to also determine CD16a expression on uNK cells, since these are in close proximity of the invading trophoblast and can directly interact with these cells during early pregnancy^36^. Although feasible protocols exist, uNK cells are more difficult to obtain and require menstrual blood or endometrium biopsy sampling^22,37^. The link between *FCGR3A*-p.176 polymorphisms and uNK cell CD16a expression levels has not been studied yet. However, previous studies showed that uNK cells predominantly lack CD16a expression and have a CD56^bright^CD16a^negative^ phenotype which is in contrast to pNK cells where 85–95% of NK cells is CD56^dim^CD16a^positive^^38–40^. Despite their low frequencies, CD16a^positive^ NK cells have been described to be present in decidua of 81.4% of women with a history of antiphospholipid antibody syndrome experiencing RPL^41^. Also, a significantly higher absolute cell count of CD16a^postive^ endometrial NK cells has been described in infertile women when compared with fertile women^42^. This suggests that CD16a may be an important marker of failed immunological adaptation during the implantation phase of early pregnancy. Furthermore, CD16a expression on uNK cells could be increased by local factors. For example, decidual NK cells have been shown to acquire CD16a expression and a more cytotoxic effector function upon interaction with cytomegalovirus (CMV) infected fibroblast^43^, and CMV seropositivity has been described to be more frequently present in women with RPL as compared to control women with a healthy pregnancy^44^. To further investigate the potential impact of *FCGR3A*-p.176 polymorphism, genotyping including CNV and analysis of CD16a expression levels on uNK cells could be combined with analysis of viral status.

Since we anticipated that the hypothesized effect of *FCGR3A* polymorphism would be most pronounced in women with alloantibodies, anti-HLA antibody status was determined. In our small cohort, we did not observe an association between HLA antibody status and frequencies of the receptor genotypes. HLA-antibodies are known to play an important role in organ transplantation as the presence of pre-transplantation donor-specific HLA-antibodies is associated with rejection and impaired organ survival^45^. In pregnancy, the presence of HLA antibodies is presumably largely a harmless phenomenon since they occur in relatively high numbers in normal pregnancy^46^. However, their role could be debated as also harmful effects of anti-HLA antibodies on pregnancy outcome have been described^47^. A higher incidence of anti-HLA antibodies has been observed in women with RPL compared to women without RPL^26^. The exact mechanism behind increased HLA antibody formation in women with RPL is currently unclear but increasing gravidity^24,48^ and the fetal and maternal HLA phenotype combination^49^ may be important determinants responsible for the higher incidence of anti-HLA antibodies in women with RPL.

In our group of women with RPL, anti-HLA antibodies were present in 81% (26 out of 32). This is higher than seen in the previous mentioned study where anti-HLA antibodies were detected in 32% of RPL cases^26^. Since none of our women had reported a previous blood transfusion or transplant in their medical history, antibodies present were most likely from a previous pregnancy. Unfortunately, HLA-typing of the partner was unknown so it was not entirely certain that the antibodies present were directed against paternal antigens of the current- or a previous conceptus. In our Luminex assays, we assigned a MFI signal of > 1000 as anti-HLA antibody positive which was based on our experience with these assays in routine diagnostics for kidney- and stem cell transplantation. However, it remains to be elucidated whether this is a clinically relevant cut off for RPL. Our cohort was too small to draw strong conclusions based on the difference between the presence of antibodies against HLA. For future analysis it would be relevant to study the presence of class I and class II antibodies in more detail as it has been shown in a transplantation setting that having HLA class I and II donor specific antibodies is a clear risk factor for graft survival in especially deceased donor transplants^28^. Having class I and II antibodies might possibly also be a risk factor for pregnancy loss in women with RPL. To study this hypothesis more thoroughly, a larger cohort of women would be necessary and follow-up of these women in necessary to see if they indeed endure more losses over a longer period of time.

In addition, there are other ADCC-inducing (allo-)antibodies, aside from HLA, that might play an important role during pregnancy. Anti-platelet antigen antibodies have been associated with pregnancy loss, as an increased maternal ADCC immune response to fetal platelet antigens has shown to cause pregnancy loss in a murine model^23^. However, we did not determine anti-platelet antigen antibodies in our study. In addition, anti-phospholipid antibodies have been highly associated with recurrent pregnancy loss^50^. The women in our cohort did not have anti-phospholipid antibodies as they were tested for during the PCVS thrombophilia screening.

To study the potential impact of humoral rejection, including ADCC, in RPL in more detail several additional factors could be included in future analysis. Antibodies can only trigger ADCC if their cognate (paternal) antigens are sufficiently expressed on fetal cells. Under physiological conditions, trophoblast cells that are in close proximity with the maternal immune system lack expression of classical HLA class Ia and class II antigens while they do express HLA class Ib antigens which are known to dampen immune responses at the feto-maternal interface^25^. The almost complete lack of expression of paternal polymorphic HLA molecules would normally protect trophoblast cells from binding to anti-HLA antibodies and thus from complement- or cell mediated cytotoxicity. Extravillous trophoblast cells do, however, express polymorphic HLA-C molecules and mismatches in highly immunogenic HLA-C*07 and -C*17 have been associated with RPL^51^. Interestingly, a higher incidence of antibodies specific for HLA-C was previously found in women with recurrent pregnancy loss illustrating that more in-depth analysis of antibody specificity may help to unravel their role in the pathophysiology of unexplained recurrent pregnancy loss. Another factor to consider is inflammatory status of the uterus, as in early pregnancy a disturbed balance between tolerogenic- and inflammatory immune reactivity may lead to aberrant expression of paternal HLA molecules contributing to humoral rejection. Although this has not been studied in the uterus yet, inflammation-induced expression of HLA has been associated with humoral rejection in the transplantation setting^52^. In addition, human islets do not express HLA class II under normal conditions, but under inflammatory conditions there is induced expression of HLA class II^53^. Increased expression of paternal alloantigens like HLA together with increased CD16a expression and a more cytotoxic profile of uNK cells could then possibly lead to ADCC in early pregnancy, while under normal conditions no ADCC would occur, and having at least one valine allele for *FCGR3A*-p.176 may aggravate the response.

In summary, with the current set of experiments, we did not observe an association between the p.Val176Phe *FCGR3A* SNP and RPL. To further investigate the role of *FCGR3A*-p.176 polymorphisms and their possible association to functional uterine NK cell ADCC activity in RPL pathophysiology, genomic analysis can be combined with more in-depth analysis of (allo)antibody profiles and viral status.

## Materials and methods

### Sample collection

Women with RPL: Blood samples of 50 women with RPL were available for genotyping the p.V176F region of *FCGR3A*. Of these 50 samples, 41 were available for additional flowcytometric analysis and 32 sera samples were available for anti-HLA antibody testing. All women gave informed consent and participated in the preconceptional cardiovascular assessment program (PCVS). The PCVS evaluation is performed according to Dutch national guidelines (www.nvog.nl) of RPL at least 3 months after pregnancy loss and consists of parental karyotypic screening, thrombophilia screening, endocrine screening and gynecological ultrasound. Women with 2 or more reported pregnancy losses before 24 weeks of gestation, were included. Women were excluded if outcomes from the PCVS evaluation indicated abnormal parental karyotype, thrombophilia (for example, presence of anti-phospholipid antibodies/Factor V Leiden mutation/Prothrombin mutation/Lupus anticoagulant or deficiency of Protein C, Protein S, antithrombin), endocrine abnormality (e.g., thyroid dysfunction) or uterine anomalies. Leukocytes were isolated from ethylene diamine tetra acetic acid (EDTA) blood samples and directly used for flowcytometry and additionally for DNA extraction using the QIAamp DNA blood mini kit (Qiagen, Hilden, Germany). This study was approved by the Medical Ethical Committee of the Maastricht University Medical Centre (MUMC +) (14-4-118) and in accordance with the Declaration of Helsinki. Information on baseline characteristics and blood samples were obtained upon written informed consent.

Healthy women: a cohort of 164 Dutch healthy female donors from Sanquin Amsterdam, that gave written consent and were genotyped for the *FCGR3A* polymorphism in the context of a previous study^54^.

### Genotyping the p.V176F region of FCGR3A

Genotyping of the 50 women with RPL for *FCGR3A* (p.V176F) was performed by sanger-based typing (SBT) using an *FCGR3A* gene-specific forward primer and a generic reverse primer, producing a 9654 bases long polymerase chain reaction (PCR) product, following a previously described protocol^55^. In addition, amplicons obtained were purified by ExoSAP-IT (Affymetrix, Santa Clara, California) and then sequenced using ABI BigDye Terminator Chemistry (Life Technologies) and an ABI 3730 sequencer (Life Technologies) with a specific forward and reverse sequencing primer. Data were analyzed using DNASTAR Lasergene SeqMan Pro (DNASTAR Lasergene, Madison, Wisconsin). Data were analyzed using Genemarker version 1.40 (Soft Genetics LLC, State College, PA).

The group of 164 healthy females, was genotyped in a previous study by a FCGR-specific multiplex ligation-dependent probe amplification (MLPA) assay (MRC-Holland, Amsterdam, The Netherlands)^54^. To ensure that both typing methods yielded comparable genotypic results, 5 women with of the current RPL cohort were genotyped by SBT and also by MLPA and this resulted in the same typing. Genetic data was tested for Hardy–Weinberg equilibrium.

### Flowcytometry

Freshly isolated leukocytes were stained with conjugated antibodies of 2uL anti-CD3 (VioBlue, REA613, Miltenyi Biotec, Germany), 2uL anti-CD56 (APC-Vio770, REA196, Miltenyi Biotec, Germany) and 2uL anti-CD16a (FITC, REA423, Miltenyi Biotec, Germany) for 30 min at 4 °C in the dark. After two wash cycles, samples were measured on a FACS Canto II (BD Biosciences, San Jose, CA) and analyzed with the BD FACSDiva Software v8.0.2 (BD Biosciences, San Jose, CA) see supplementary file for gating strategy. In order to reduce inter-experimental variations, all samples were measured at a standardized sample rate of 5000 NK cells and with application settings to standardize voltage and compensation settings. Variation from the background signal was abrogated by normalizing MFI values with a FMO, see Fig. 3G for representative histogram.

### Anti-HLA antibody testing

Serum anti‐HLA antibodies were tested using single antigen beads (LABScreen, One Lambda Inc/ThermoFisher, Canoga Park, CA), according to the manufacturer’s instructions. Anti‐HLA antibody profiles were analyzed using the HLA Fusion software v4.2 (One Lambda Inc/ThermoFisher, Canoga Park, CA). Antibodies with a mean channel fluorescence intensity (MFI) lower than 1000 were considered negative and antibodies with an MFI higher than 1000 positive.

### Statistical analysis

Data was tested for normality with Shapiro–Wilk. Dichotomous data was analyzed with Chi Square and was compared between women with RPL and an independent cohort of healthy women (percentages of frequencies of polymorphisms) and between women with RPL categorized as either being positive or negative for class I, class II and class I and/or class II anti-HLA antibodies and between women with RPL categorized as only having class I, class II, class I and II or no anti-HLA antibodies (percentages of frequencies of polymorphisms), respectively. Continuous data among women with RPL was analyzed with Mann Whitney U or Kruskal–Wallis (percentages of NK cells (CD3^negative^CD56^positive^), and of the CD56^dim^CD16a^positive^ and CD56^bright^CD16a^negative^ NK cell subsets and percentages plus MFI of CD16a expression on NK cells). Overall, a P-value below 0.05 was considered statically significant and all statistical analyses were conducted with IBM SPSS statistics version 25 (IBM Corp, Los Angeles, USA).

## Supplementary Information

Supplementary Information.

## Data Availability

The datasets used and/or analyzed during the current study available from the corresponding author on reasonable request.
