# Supplementary material for: Analysis of FCGR3A-p.176Val variants in women with recurrent pregnancy loss and the association with CD16a expression and anti-HLA antibody status
Source: Sci Rep. 2023 Mar 30;13:5232. doi: 10.1038/s41598-023-32156-9 (PMC10063683; doi:10.1038/s41598-023-32156-9)
Supplement: Supplementary file 1 — Supplementary Information. [file 41598_2023_32156_MOESM1_ESM.pdf]

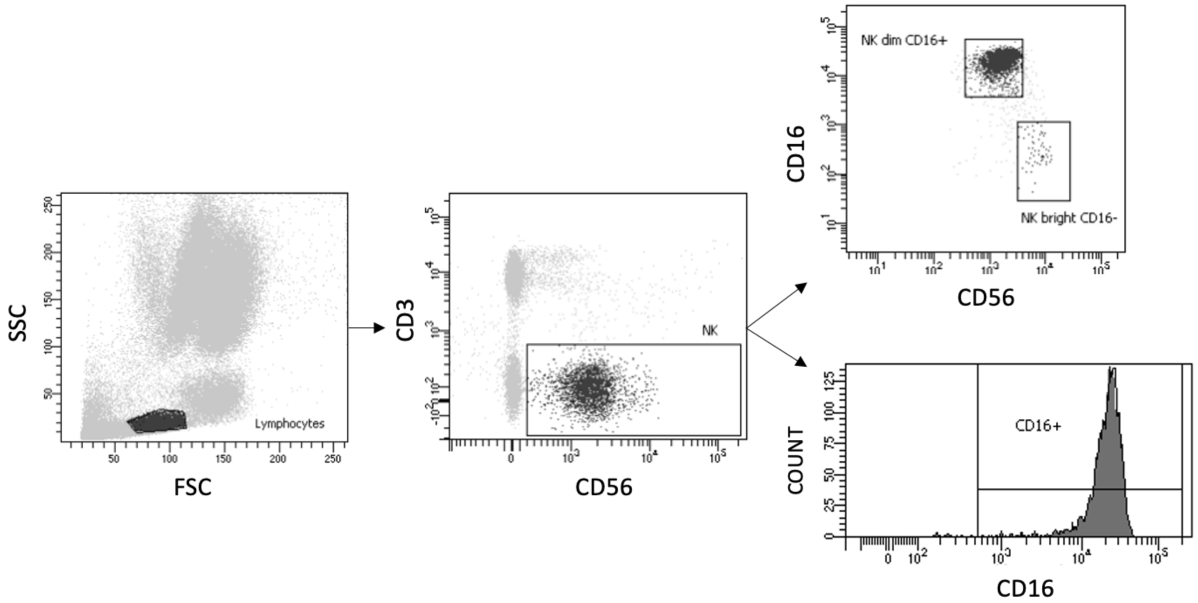

FigureS1: Representative flow cytometric gating strategy to identify NK cells in peripheral blood of women with RPL. NK cells were gated as CD3^{negative}CD56^{positive} and delineated by expression of CD16.
